# Reviewed article: Mori MM, Piran CMG, Cargnin AVE, Moroskoski M, Fernandes CAM, Moura DRO, Furtado MD.Geographical distribution and spatial autocorrelation of newborns with orofacial clefts: an epidemiological study, Paraná, 2018-2022. Epidemiol Serv Saude. 2025:34;e20250189

**DOI:** 10.1590/S2237-96222025v34e20250189.a

**Published:** 2025-10-31

**Authors:** José Mauro de Oliveira Squarisi

**Affiliations:** 1 Universidade Federal de Uberlândia, Uberlândia, MG, Brazil Universidade Federal de Uberlândia Uberlândia MG Brazil

## Peer review A

## Questionnaire

Does the manuscript contain new and significant information to justify publication? Yes

Does the Abstract (Summary) clearly and accurately describe the content of the article? Yes

Is the problem significant and concisely stated? No

Are the methods described comprehensively? Yes

Are the interpretations and conclusions justified by the results? No

Is adequate reference made to other work in the field? No

## Manuscript Structure

Length of article is: Adequate

Number of tables is: Too few

Number of figures is: Adequate

Please state any conflict(s) of interest that you have in relation to the review of this paper. None

Interest: Good

Quality: Below Average

Originality: Good

Overall: Average

Recommendation: Major Revision

## Comments

O desenho do estudo permite inferir as regiões com maior número de diagnósticos, porém algumas informações (como por exemplo a exposição a agrotóxicos, exposições ambientais não podem ser inferidas por este tipo de estudo.

Comparações genéricas também foram realizadas (por exemplo cidades na África do Sul com a cidade portuária de Paranaguá) não sendo possível confirmar esta relação.

Algumas informações foram realizadas de maneira genérica (por exemplo: Pesquisa realizada no sul do Irã apresentou muitos casos de fissuras lábio palatinas. Muitos? Quantos? Qual a taxa?).

O primeiro e metade do segundo parágrafo da discussão seria melhor alocado nos resultados (A auto correlação espacial... até Cornélio Procópio/Bandeirantes).

